# Sucrose supplementation influences gut microbial diversity and functional shifts in *Apis cerana indica*

**DOI:** 10.3389/fmicb.2025.1733283

**Published:** 2026-01-22

**Authors:** Deeptismita Nayak, Pravasini Behera, Satyapriya Singh, Partha Sarathi Tripathy, Soumya Shephalika Dash, Madhumita Dasgupta, Sansuta Mohanty, Manas Ranjan Sahoo, Chitta Ranjan Satapathy, Arundhati Sasmal, Subhasmita Sahu

**Affiliations:** 1 Department of Entomology, College of Agriculture, Odisha University of Agriculture and Technology, Bhubaneswar, Odisha, India; 2 Central Horticultural Experiment Station, ICAR-Indian Institute of Horticultural Research, Bhubaneswar, Odisha, India; 3 Department of Fisheries Resource Management, College of Fisheries, Rani Lakshmi Bai Central Agricultural University, Datia Campus, Datia, Madhya Pradesh, India; 4 Department of Agricultural Entomology, Institute of Agriculture, Visva-Bharati, Sriniketan, West Bengal, India; 5 Department of Molecular Biology and Biotechnology, Institute of Agricultural Sciences, Siksha O Anusandhan, Bhubaneswar, Odisha, India; 6 ICAR-Central Tuber Crops Research Institute, Thiruvananthapuram, Kerala, India; 7 Department of Floriculture and Landscaping, College of Agriculture, OUAT, Bhubaneswar, Odisha, India

**Keywords:** *Apis cerana indica*, gut microbiota, metagenomics, microbial diversity, sucrose supplementation

## Abstract

Honeybee colonies are increasingly threatened by nutritional scarcity and biotic stressors, underscoring the need to understand the role of gut microbiota in mitigating these challenges. This study examined the gut microbial composition of *Apis cerana indica* under two dietary regimes: sucrose–fed and sucrose–unfed, to assess how nutrition influences microbial diversity and metabolic potential following metagenomics. Metagenomic sequencing of gut samples revealed 147,146 contigs, with the longest and shortest contigs measuring 615,154 kb and 200 kb, respectively. Comparative analysis indicated a higher relative abundance of *Bacillus* spp. in sucrose–fed bees, whereas *Enterococcus* was more dominant in unfed populations. Sucrose feeding significantly enhanced gut microbial diversity (Shannon index: 2.59; Simpson’s index: 0.87) compared to unfed bees (Shannon: 1.91; Simpson: 0.68). Key genera, including *Gilliamella*, *Bacillus*, and *Lactobacillus*, were consistently present but showed varying relative abundances. Functional annotation via KEGG pathway analysis revealed elevated activity of glycolysis and the pentose phosphate pathway in sucrose-fed bees, with exclusive detection of key metabolic enzymes, hexokinase and enolase. Additionally, elevated sucrose metabolism and proteolytic enzyme activity were noted, reflecting enhanced metabolic versatility. Our findings highlight the importance of sucrose dietary supplementation in shaping gut microbial structure and function, their diversity, and metabolic capacity, suggesting its potential as a practical nutritional intervention to sustain honeybee health during a period of floral dearth. The outcome of the study encourages exploring the long–term ecological and physiological impacts of dietary strategies on colony resilience and productivity.

## 1 Introduction

The Indian honeybee, *Apis cerana indica* Fabricius, plays a vital role in biodiversity conservation and agricultural productivity by facilitating the pollination of a wide range of cultivated and wild plant species. *A. cerana indica* acts as a social insect and has successfully established across diverse ecosystems globally. In India, they contributed more than 33,128 million by 2024, expanding at a Compound annual growth rate (CAGR) of nearly 12% by 2024 to the agricultural industry annually (NITI Aayog, 2024). However, the bee population has been declining alarmingly due to various factors, including habitat fragmentation, pesticide exposure, and high parasite and pathogen loads (Formentin, 2022). While various management strategies have been explored to mitigate these threats, nutritional supplementation remains a key area of research. Feeding the sugar solution, preferably sucrose, to honey bee colonies is a valuable need-based management tool for beekeepers (Abdella et al., 2024). Despite the widespread use of sucrose solutions as a supplemental feed for honey bee colonies (Brodschneider and Crailsheim, 2010), there is limited understanding of its long-term effects on colony health, immunity and foraging behavior (Ghosh et al., 2020). Most studies have focused on the immediate benefits of sucrose feeding, such as stimulating foraging activities, supplementing stored honey shortages, and preventing colony starvation (El Ghouizi et al., 2023). However, the potential tradeoffs between artificial feeding and natural dietary sources and their impact on overall bee resilience require further investigation and in depth understanding.

The gut microbiome constitutes a complex and dynamic ecosystem comprising diverse microbial communities (Suzuki et al., 2022). Its composition and abundance are modulated by multiple factors, including host phylogenetic background (Seedorf et al., 2014), dietary inputs (Groussin et al., 2017), endocrine signaling, social behavior (Zhang et al., 2022), and antibiotic exposure (Patangia et al., 2022). Further, the gathered evidence depicts that the gut microbiota of adult honeybees participates significantly in sucrose digestibility for honey bee health (Moran, 2015). The bee microbiomes transmute dietary compounds and fabricate short–chain fatty acids in the gut, which helps in enhancing sucrose responsiveness and revitalizes the immune system. Due to the hyper flexibility of bacterial gene content, the functions of these communities depend on their strain-level composition as well as the supplemental nutrition. Gut microbes are important in bee health and disease (D’alvise et al., 2018).

Several studies have evidenced the composition of gut microbiota in different worker bee types throughout seasons and broadly concluded that the overall community composition is relatively stable. However, previous studies were primarily based on comparative analyses of relative abundances by using 16S rRNA gene amplicon sequencing (Ellegaard et al., 2020). Analyses like those failed to provide significant insights about the extent or way of abundance changes, especially if microbial loads vary substantially between varied samples of different treatments. A striking feature of the honeybee gut microbiota is its low taxonomic complexity. In worker bees, the community is dominated by less than ten phylotypes, which typically make up more than 95 per cent of the bacterial cells in the gut (Ellegaard and Engel, 2019). They include some core phylotypes like *Gilliamella, Snodgrassella, Lactobacillus,* and *Bifidobacterium*, which are typically present in every adult worker bee (Kwong and Moran, 2016). These phylotypes have been consistently detected in honeybees, regardless of geographic location, life stage, or season and are acquired horizontally through contact with nest mates and hive components (D’Alvise et al., 2018; Jones et al., 2018). Moreover, the physiological and microbial health of bees, particularly their gut microbiota, is another underexplored frontier. The balance of beneficial microorganisms in the bee gut is essential for its digestive efficiency and immune defense against pathogens. Understanding these interactions at a microbial level offers valuable insights into how artificial feeding may inadvertently exacerbate the challenges faced by honey bee populations worldwide (Liu et al., 2022).

Metagenomics refer to the study of the genetic materials of the organisms in a specific environment, representing the entire communities without culturing the individual microbes. A recent report focused on gut microbiota community analysis through metagenomics (Hariprasath et al., 2025; Tran et al., 2025). However, studies on the influence of artificial sucrose feeding on gut microbiome are scanty. A comprehensive investigation into the cumulative impact of artificial feeding on honey bee health over multiple generations is essential to formulating sustainable management strategies that balance the need for supplemental nutrition with preserving bees’ natural foraging and nutritional behaviors (Frizzera et al., 2020). Abridging the research gaps surrounding sucrose feeding and honey bee health is critical for developing effective, ecologically sound strategies for bee management. This research has far-reaching implications not only for improving bee health and resilience but also for safeguarding the essential ecosystem services that honey bees provide, particularly in agricultural and natural landscapes. By addressing the unanswered questions in this area, this study hypothesized that supplemental sucrose feeding would significantly alter the gut microbial composition and functional potential in *Apis cerana indica*, thereby influencing key metabolic pathways and immune responses. The investigation aimed to comprehensively evaluate the diversity and structure of gut microbiota in *A. cerana indica* under natural and sucrose-fed conditions using metagenomics tools. Additionally, it sought to functionally annotate these microbial communities through KEGG (Kyoto Encyclopedia of Genes and Genomes) based metabolic pathway analysis to uncover diet-induced variations. By identifying core microbial taxa and key functional genes affected by feeding practices, the study intended to provide deeper insights into the nutritional ecology of honeybees, with broader implications for sustainable bee management strategies. Moreover, this study focused on the diversity that enriches the gut microbiota of *Apis cerana indica* and also carried a comprehensive analysis of the associated KEGG metabolic pathways, incorporating the effect of metabolic alteration in different dietary conditions. We propose that understanding the dietary variation, i.e., natural and supplemental sucrose feeding, will contribute significantly to advancing the management of honey bees during the period of scarce resources in nature. This study employed metagenomics to investigate the structural and diversity differences in the gut microbiota of *A. cerena indica* under sucrose-fed and sucrose-unfed conditions. Henceforth, understanding this microbial community will provide valuable insights into host–microbe interactions, contributing to the development of strategies for enhancing honeybee health and optimizing apicultural practices (Hroncova et al., 2019). Furthermore, these findings implicate new questions about the functional potential and evolutionary history of host-associated bacteria and their influence on honeybee health.

## 2 Materials and methods

### 2.1 Colony maintenance

*Apis cerana indica* colonies were maintained with the help of desirable requisites hives as per the Indian Standards Institute (ISI) type ‘A’ (W29 X L36 X H41 cm and 60 cm height above the ground) in the experimental location at the Medicinal Plants Knowledge Centre, Bhubaneswar, India (located at 85.757427° E, 20.245297° N and 26 m above mean sea level altitude). A total of 24 honey bee colonies were maintained for 1 year in two different treatments, i.e., with sucrose feeding (2:1 sucrose and water) once a week and without sucrose feeding (water). The feeding (250 mL) was given with the help of containers, preferably between 3 and 4.30 p.m.

### 2.2 Specimen collection

Healthy foraging workers of *A. cerena indica* were collected at the entrance of hives from the apiary at the experimental site. One hundred adult workers for each treatment were collected from five different hives using an insect net between 8 and 10 a.m. during April 2024. Bees were anaesthetized using benzene and cleaned with water. Further, a surface sterilization was done with 70% ethanol to remove the external microorganisms or contaminants. A sterile needle was used to fix the worker’s thorax, and pinned onto a wax surface. We used sterile micro forceps and pulled out the entire alimentary canal carefully in one smooth flow. The pooled bees’ guts were placed in a 2 mL centrifuge tube with 10x saline PBS solution, respectively (Coleman et al., 2007). The samples were kept at −80^0^ until the DNA extraction.

### 2.3 Isolation of genomic DNA

The genomic DNA of insect gut samples were extracted using DNeasy Blood and Tissue Kit (Qiagen, New Delhi, India) following the manufacturer’s instructions. Lysis buffer was added to a mixed zirconium bead tube containing the gut sample. Bead beating has been carried out using a standard benchtop vortex with a bead tube adapter. Crude lysate is then subjected to inhibitor removal for clean-up. Following the clean-up, purified lysate was mixed with an equal volume of DNA binding solution and passed through a silica spin filter membrane. The membrane was washed with a two-step washing regime, and DNA was extracted. The concentration of DNA was checked using a Qubit 3.0 fluorometer (Thermo Fisher Scientific, Waltham, MA, USA) using a DNA HS assay kit (Thermo Fisher Scientific, Waltham, MA, USA). DNA purity was checked using Nanodrop 2000 (Thermo Fisher Scientific, Waltham, MA, USA). The integrity of DNA was evaluated on 1% agarose gel (Lonza, Belgium).

### 2.4 Short gun metagenome library preparation

We took 350 ng of DNA as an initial input for starting the library. We diluted the samples according to the concentration using the KAPA Hyper Plus Kit (KAPA, Pleasanton, CA, USA) as per the manufacturer’s protocol. The library started with the fragmentation of the genomic DNA, followed by end repair and tailing the fragments, which was followed by adaptor ligation and amplification of the fragments. The prepared libraries were quantified using a Qubit 3.0 fluorometer (Thermo Fisher Scientific, Waltham, MA, USA) using a DNA HS assay kit (Thermo Fisher Scientific, Waltham, MA, USA). The size of DNA was checked on Agilent 2,100 bioanalyzer using Agilent high sensitivity DNA assay kit (Agilent high sensitivity DNA assay kit, Lab India, Thane, Maharashtra, India).

### 2.5 Gut microbiome sequencing

Sample pooling was performed based on fragment size and concentration to meet the required sequencing depth. The pooled libraries were then sequenced using the Illumina NovaSeq 6,000 platform (Illumina, San Diego, CA, USA) with an S4 flow cell. The gut microbiome metagenomics sequencing and data processing were performed at M/S Nucleome Informatics, Hyderabad, India.

### 2.6 Metagenomic data processing, assembly, and annotation

The raw reads of samples were subjected to adapter trimming with fastp, and the quality of adapter-trimmed reads was checked using FastQC (V 0.12.0, accessed in August 2024). Metagenome assembly was performed on clean reads of all samples using the MEGAHIT assembler (Version 1.2.9, accessed in August 2024). Metagenome Assembled Genomes (MAGs) were extracted from the assembly by a process called ‘binning’ for both samples. The bins extracted from the assemblies were refined further to obtain MAGs which are complete in terms of completeness with lesser contamination. Taxonomic classification and functional annotation were performed for the MAGs using Diamond software (Version 5.1, accessed in August 2024).

### 2.7 Functional analysis of feeding vs. non–feeding samples

The quality-filtered reads were analyzed using the SqueezeMeta pipeline (Version 1.6.2 accessed in August 2024), an automated tool for metagenomics and metatranscriptomic analysis. The pipeline was run in co-assembly mode, which allowed the combined assembly of feeding and non-feeding datasets using MEGAHIT (Version 1.2.9, accessed in August 2024). Gene prediction was performed using Prodigal, and taxonomic annotation was carried out by comparing predicted proteins against the NCBI NR database using DIAMOND (Version 5.1, accessed in August 2024). Functional annotations were obtained through searches against eggNOG, KEGG, and COG databases. Read mapping was done using Bowtie2 (Version 2.5.4), and gene abundance quantification was conducted using feature Counts.

For enhanced visualization and bin refinement, SqueezeMeta outputs were imported into Anvi’o using the provided integration script. Anvi’o was used to inspect taxonomic and functional composition, refine contig binning, and explore metagenome-assembled genomes (MAGs) when relevant. Interactive plots were generated to investigate the distribution and abundance of contigs and functions across feeding states. Further downstream statistical analysis and visualization were carried out in R using the SQMtools package. The SqueezeMeta project was loaded using the loadSQM function. Gene and taxonomic abundances were normalized using transcripts per million (TPM) and copy-number scaling. Alpha diversity (Shannon and Simpson indices) (Shannon, 1948, Simpson, 1949) and beta diversity (Bray–Curtis dissimilarity) were computed to assess microbial community variation between the feeding and non-feeding groups. Differential abundance analysis was performed using DESeq2 (Version 1.24.0, Accessed in August 2024) using an adjusted *p*-value≤0.05, identifying significant taxa and functional genes. To assess functional differences in metabolism, log_2_ fold–change in gene abundance was specifically analyzed for three KEGG pathways: pyruvate metabolism, pentose phosphate pathway, and glycolysis/gluconeogenesis, showing enriched functions in each condition. Additionally, top KEGG Orthology (KO) functions and top COG categories were visualized through heat maps generated via SQMtools (Version 1.7.2, accessed in August 2024). Krona plots were also produced from SqueezeMeta outputs to provide an interactive visualization of the taxonomic hierarchy in each sample group.

## 3 Results

### 3.1 Taxonomic profiling of the core gut microbiota associated with *Apis cerana indica*

We examined a comprehensive profiling of the gut microbiota in sugar-feeding and non-feeding worker honey bees of *A. cerana indica*. The metagenomics analysis revealed that the total number of contigs was 147,146 kb for both samples (Table 1). The ORF (Open reading frame) statistics, annotation details, and the bins statistics of Gut microbiota have been provided in the Supplementary Tables S1–S3. Our result implied that the total length of gut microbiota was 153,085,914 kb, whereas the longest and shortest contig was found to be 615,154 kb, and 200 kb, respectively. The rank of hierarchical groups was summarized in Table 1. Our result found that contigs at phylum (p) rank were 85,049 kb (57.8%), which comprised contigs at order (o) rank 82,025 (55.7%), in 89 orders, and 22 phyla. Furthermore, the contigs at class (c) rank were about 84,353 kb (57.3%), constituting 37 classes. Moreover, contigs at order (o) rank 82,025 (55.7%) having 89 orders were ranked by family (f) genus (g), species (s) of 76,514 (52.0%) in 111 families, 74,369 (50.5%) in 203 genera, 15,880 (10.8%), in 192 species, respectively.

**Table 1:** Contig statistics of *A. cerena indica g*ut microbiota.

Particulars	Assembly
Number of contigs	147,146
Total length	153085914.00
Longest contig	615,154
Shortest contig	200
N50	2,514
N90	368
Contigs at superkingdom (k) rank	87,292 (59.3%), in 4 superkingdoms
Contigs at phylum (p) rank	85,049 (57.8%), in 22 phyla
Contigs at class (c) rank	84,353 (57.3%), in 37 classes
Contigs at order (o) rank	82,025 (55.7%), in 89 orders
Contigs at family (f) rank	76,514 (52.0%), in 111 families
Contigs at genus (g) rank	74,369 (50.5%), in 203 genera
Contigs at species (s) rank	15,880 (10.8%), in 192 species
Congruent	146,551 (99.6%)
Disparity >0	596 (0.4%)
Disparity > = 0.25	445 (0.3%)

### 3.2 Diversity study of gut microbiota of *A. cerana indica*

**Figure 1:**
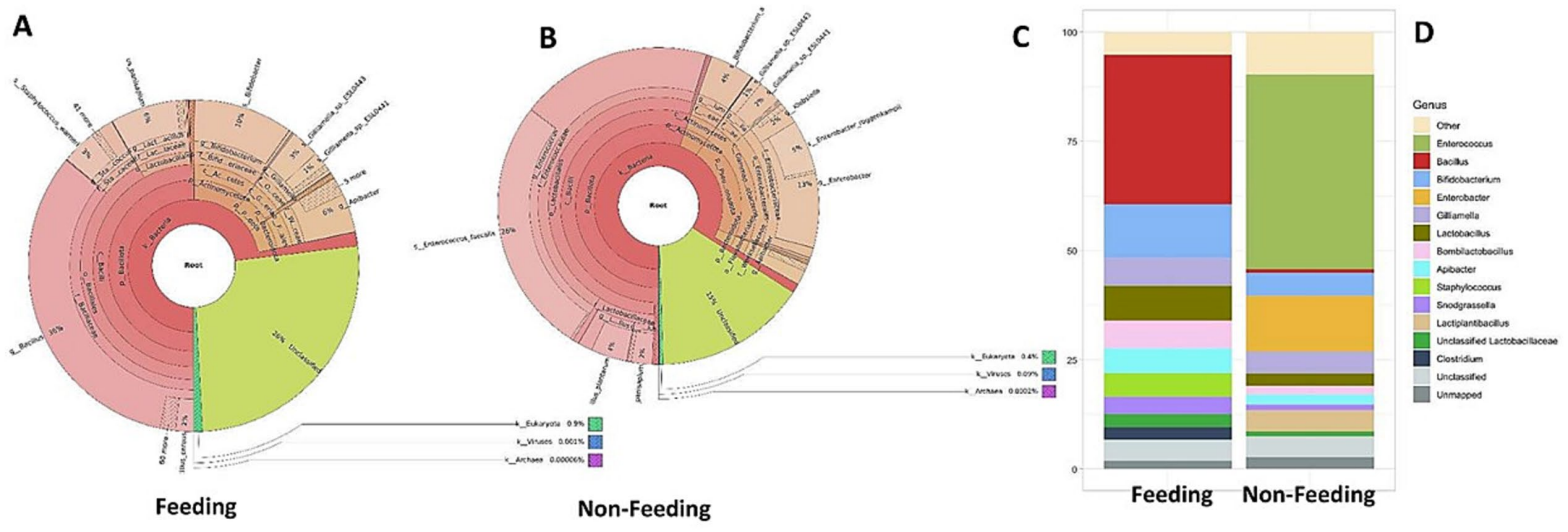
Krona plot (**A**: feeding, **B**: non-feeding) showing the mean taxa relative abundances at the phylum and genus levels of the gut microbiota of *A. cerena indica*, the stack bar showing comparison of genus level abundance in both conditions (**C**: feeding, **D**: non-feeding).

The present findings implied that the gut microbiota diversity in *A. cerana indica* for both the groups (feeding and non-feeding) was distinct and varied for relative abundance (Figures 1A–D). To demarcate an initial broad differentiation of the gut microbiota composition, the relative abundance of all genera in the database was quantified, based on mapped read coverage. The genus-level configuration comprises a total of twelve genera that have been proposed to constitute the core microbiota of bees. They all were found to colonize in both treatments, although the relative abundance arrangement profiles were discrete between them (Figures 1C,D). The genus associated with the feeding treatment of *A. cerena indica* were *Gilliamella* (4%), *Bacillus* (36%), *Snodgrassella* (6%)*, Bifidobacterium* (10%), *Lactobacillus* (6%)*, Bombilactobacillus* (5%)*, Apibacter* (6%) *Staphylococcus* (3%)*, Clostridium* (4%), and 26% of the genus remained unclassified (Figures 1A–D). On the flip side, the microbiome composition of the sucrose non-feeding group was *Gilliamella* (3%)*, Bacillus* (4%)*, Snodgrassella, Bifidobacterium* (4%)*, Lactobacillus* (4%)*, Bombilactobacillus, Apibacter* (6%)*, Klebsiella* (2%), *Enterococcus* (26%)*, Enterobacter* (13%), *Lactiplantibacillus* and 15% remained unclassified (Figures 1A–D). Our findings revealed that *Enterococcus, Enterobacter* and *Lactiplantibacillus* were not found in feeding samples of the *A. cerena indica*. Moreover, we found that eight genera were common in both the feeding and non-feeding groups, but their density varied significantly (Figures 1C,D).

Furthermore, we analyzed the diversity indices for the gut microbiome of *A. cerana indica* for both feeding and non-feeding populations using Shannon-Weaver and Simpson diversity indices. The diversity comparison revealed that Shannon’s index was 2.59 in the feeding population in comparison to 1.91 in non-feeding population. The Shannon-Weaver diversity index usually ranges from 1.5 to 3.5. The higher the value, the community is more diverse. Furthermore, we computed the Simpson index for a measure of dominance (*λ*). The range 0.61–0.8 suggests a moderately high degree, and 0.81–0.99 a high degree of diversity. We found the Simpson’s diversity was 0.87 and 0.68 for feeding and non-feeding groups, respectively. Our results implied that the feeding samples have higher diversity than the non-feeding samples.

### 3.3 Anvi’o analysis

**Figure 2:**
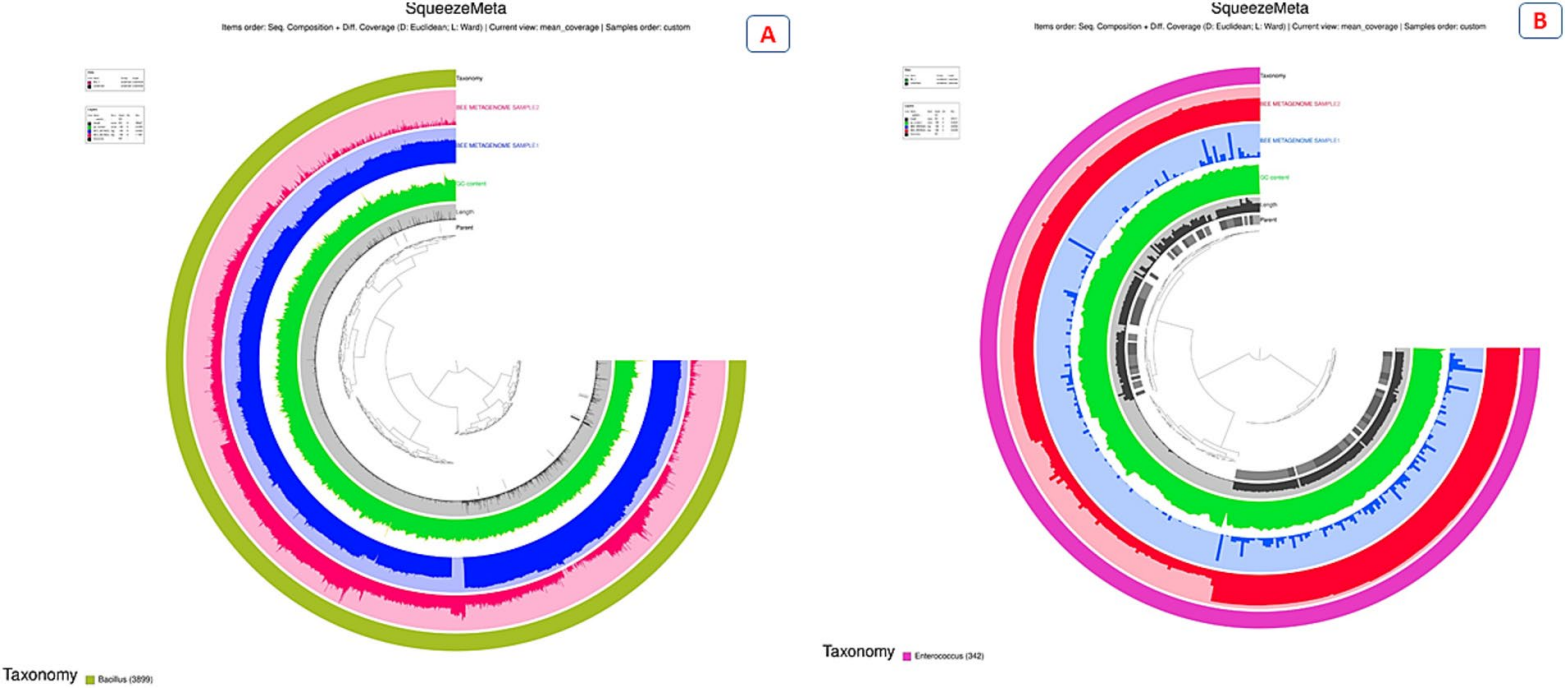
Circular plot of *Bacillus* spp. **(A)** and *Enterococcus* **(B)**.

The Anvi’o analysis of the *Bacillus* genus in feeding and non-feeding honeybee metagenome samples revealed significant differences in contig distribution and abundance (Figure 2A). The presence of differentially colored contigs suggested variations in the abundance of *Bacillus* species between the two conditions (Figure 2A). It was clearly observed that the contigs for *Bacillus* were enriched in feeding as compared to non-feeding samples overall. Additionally, some contigs have undefined values, suggesting potential gaps or unclassified sequences in the dataset. These differences in contig abundance indicated that the microbial composition, particularly within the *Bacillus* genus, varies between the two honeybee metagenomes.

**Figure 3:**
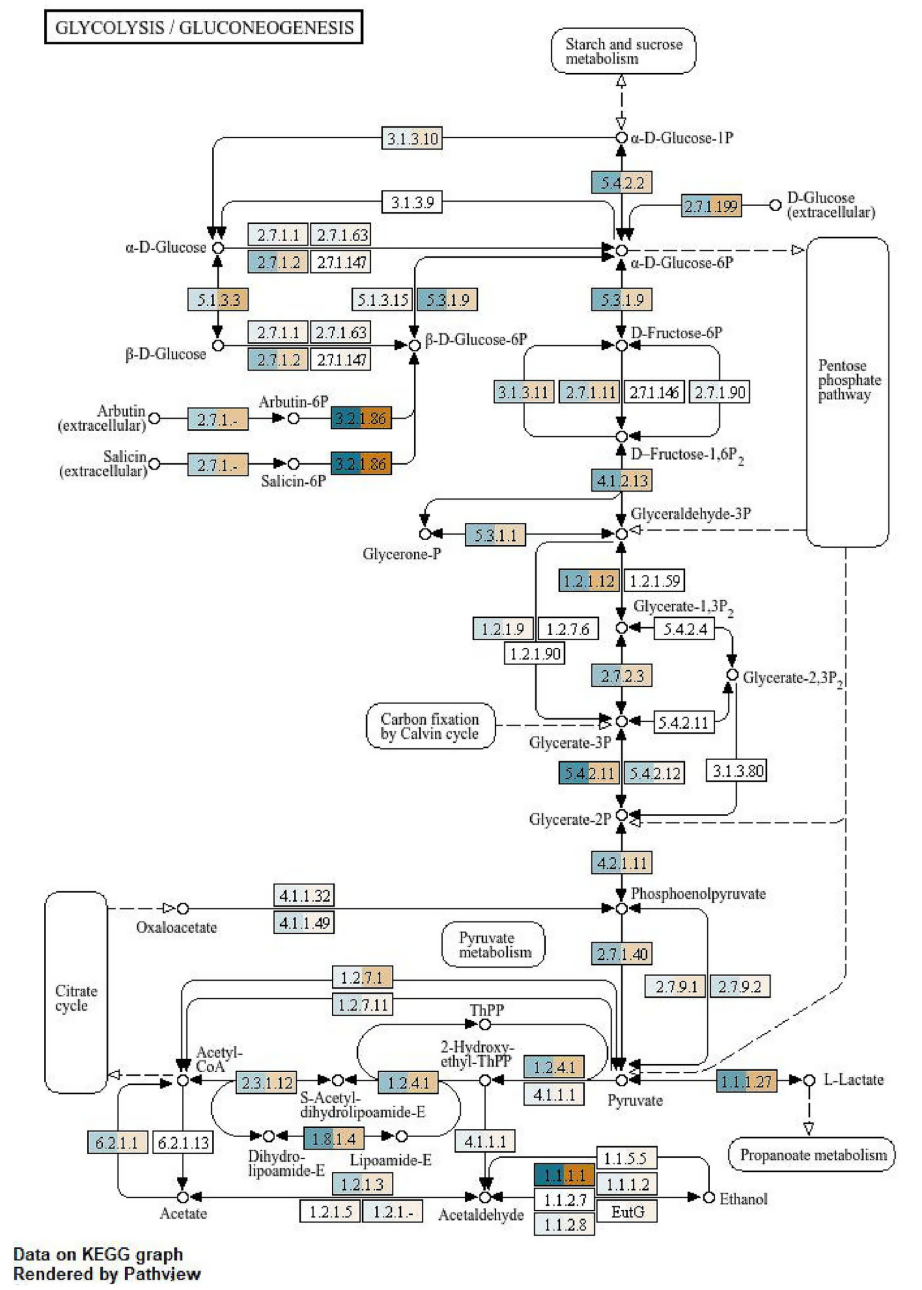
KEGG pathway on glycolysis of the gut microbiota of *A. cerena indica.* **(A)** Feeding (blue); **(B)** non-feeding (orange).

The Anvi’o analysis of the genus *Enterococcus* in feeding and non-feeding honeybee metagenome samples unfolds pronounced differences in contig distribution and abundance (Figure 3B). The presence of separately colored contigs in Figure 2B depicted variations in the abundance of *Enterococcus* species between the two samples. It was quite evident that the contigs for *Enterococcus* were enriched in non-feeding as compared to feeding samples overall. The peaks are elevated in non-feeding sample. In contrast to that, the feeding conditions have absolutely negligible traces of contigs in this genus, giving rise to new potential areas for research. These differences in the presence of distinct contigs indicated that the microbial composition was not the same, particularly between the two treatment metagenomes, i.e., with and without feeding.

### 3.4 KEGG pathway analysis

**Figure 4:**
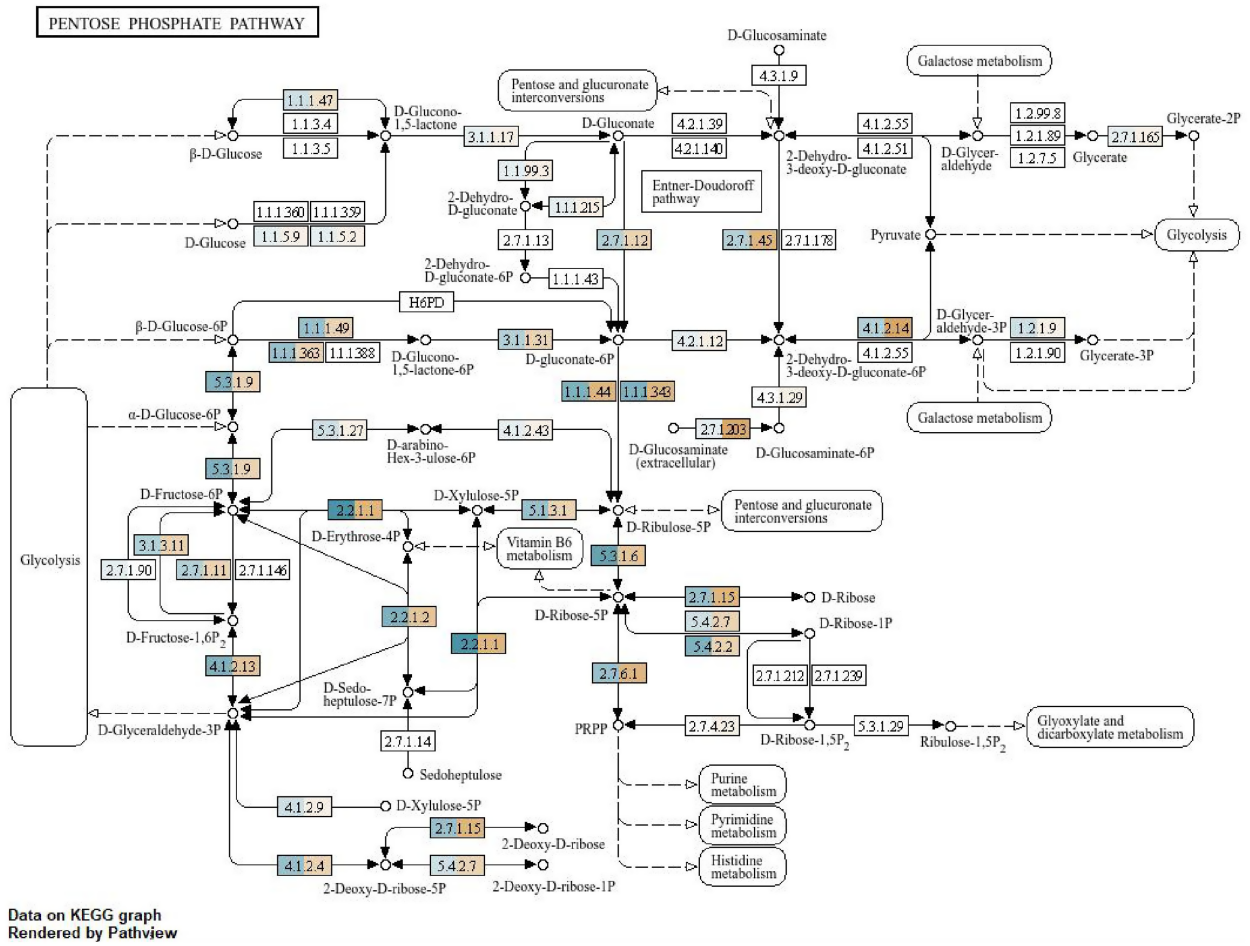
KEGG pathway on pentose phosphate pathway of the gut microbiota of *A. cerena indica.* **(A)** feeding (blue), **(B)** non-FEEDING (orange).

**Figure 5:**
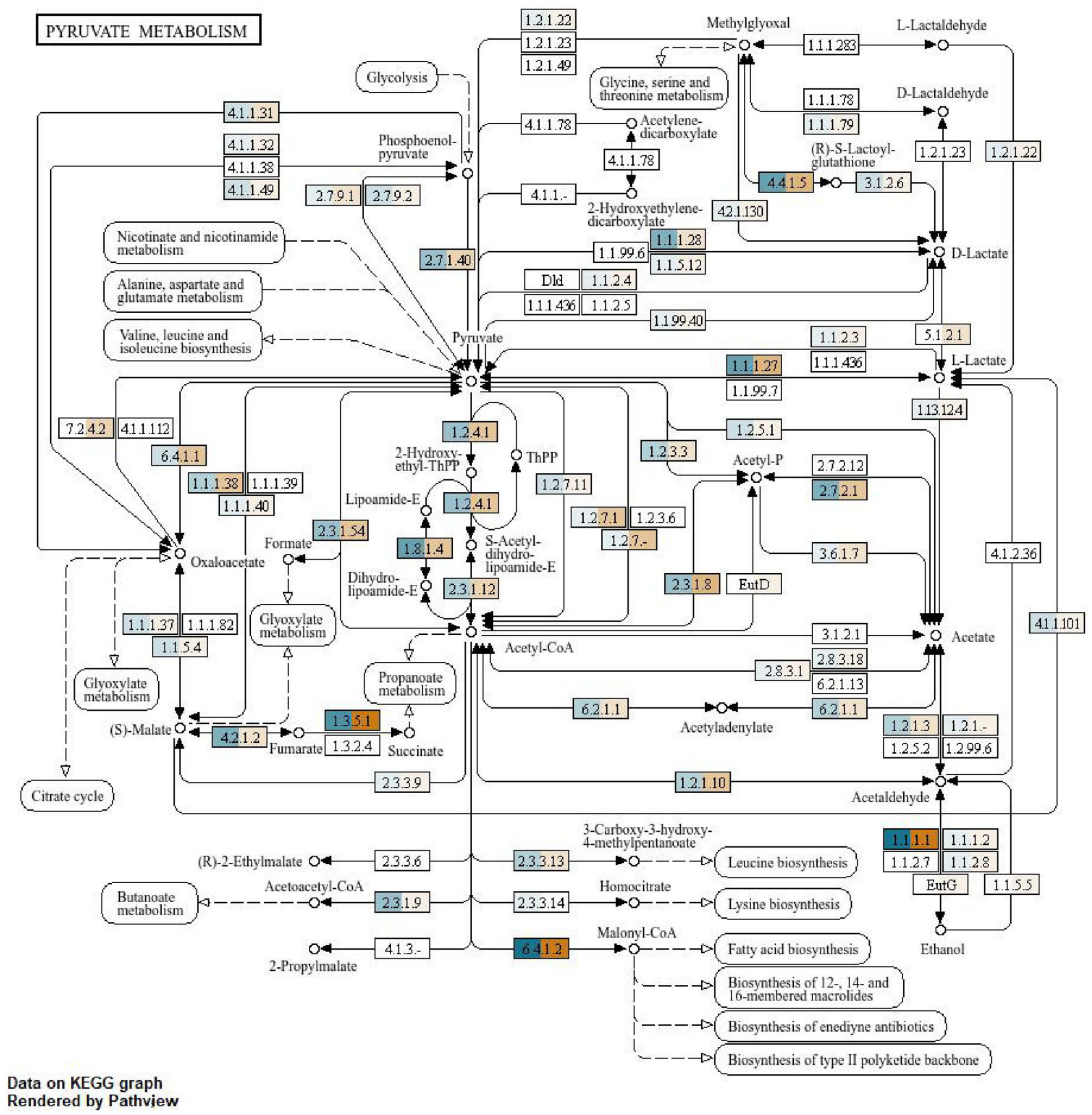
KEGG pathway on pyruvate pathway of the gut microbiota of *A. cerena indica*. **(A)** Feeding (blue); **(B)**, non-feeding (orange).

**Figure 6:**
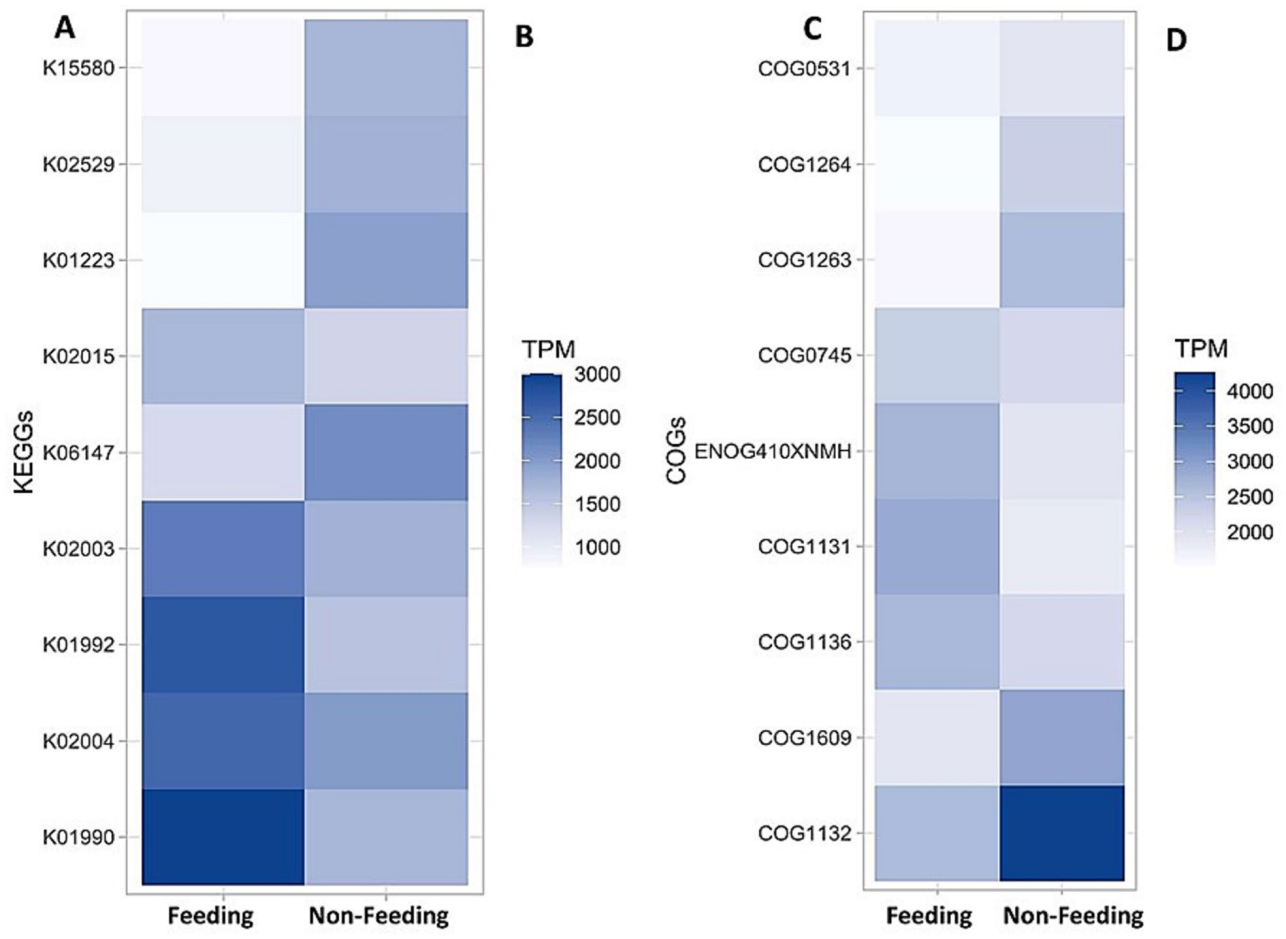
KEGG–proteins heatmap (**A**: feeding, **B**: non-feeding) and cluster of orthologous genes (**C**: feeding, **d**: non-feeding) of the gut microbiota of *A. cerena indica.*

The KEGG platform is a manually curated, harmonized database source amalgamating discrete biological entities categorized into systems, genomic, chemical and health information. Every database entry is picked out with the help of the KEGG identifier. The KEGG pathway map viewer, the Brite hierarchy viewer and the newly released KEGG genome browser are the components that have been used to analyze our data. The KEGG pathway maps representing molecular wiring pictorial representation of honey bee systems were inculcated in our study to further establish the functional aspects of the molecules that are found to be associated with sucrose metabolism. Every pathway map was examined with the specialized pathway map viewer along with an improvised mechanism for KO (KEGG Orthology) assignment. The differentiation of two treatments for functional annotation of the enzymes and chemical compounds was described (Figures 3–6). The whole process was conducted using SqueezeMeta and SQMtools in R, revealing significant differences in the presence of metabolic enzymes between feeding and non-feeding conditions. The blue–colored enzymes depicted those ascertained in the feeding condition, while the orange–colored enzymes disclose those present in the non–feeding condition.

### 3.5 KEGG glycolysis pathway analysis

The KEGG glycolysis/gluconeogenesis pathway analysis of the bee metagenome was conducted, and the result revealed significant differences in the presence of metabolic enzymes between feeding and non-feeding conditions. Several key glycolytic enzymes were found exclusively in the feeding condition, suggesting an active glycolysis pathway that facilitated the efficient breakdown of glucose for energy production. Additionally, Hexokinase (EC 2.7.1.1 & 2.7.1.63) along with phosphoglucomutase was detected, that is responsible for converting glucose–1–phosphate into glucose-6-phosphate, a crucial process in carbohydrate metabolism. Further along the pathway, glyceraldehyde-3-phosphate dehydrogenase (EC 1.2.1.12) was observed in feeding bees, enabling the conversion of glyceraldehyde-3-phosphate into 1,3-bisphosphoglycerate, a key energy-yielding step. Similarly, enolase (EC 4.2.1.11) was detected, facilitating the transformation of 2-phosphoglycerate into phosphoenolpyruvate, an essential precursor for ATP generation (Figure 3). These findings suggested that bees in the feeding condition relied primarily on glycolysis for immediate energy production and metabolic activity.

In contrast, non-feeding bees exhibited a metabolic shift towards alternative energy production pathways, as reflected in the exclusive presence of enzymes associated with gluconeogenesis, fermentation, and anaerobic metabolism. Pyruvate ferredoxin oxidoreductase (EC 1.2.7.1 & 1.2.7.11) was detected in non-feeding bees, indicating a reliance on anaerobic pathways to utilize pyruvate when glucose availability was limited (Figure 3). Additionally, the presence of alcohol dehydrogenase (EC 1.1.1.1 and 1.1.1.5) suggested an increased dependence on ethanol fermentation, potentially as a survival strategy under nutrient-deficient conditions. Furthermore, phosphoenolpyruvate carboxykinase (EC 4.1.1.32) was present, indicating an active gluconeogenesis pathway that enabled the conversion of oxaloacetate into phosphoenolpyruvate, allowing glucose production when external dietary sources were scarce.

### 3.6 Pentose phosphate pathway analysis

The functional annotation of gut microbiota in the KEGG database was investigated, and these annotated biological functions were categorized. The KEGG pentose phosphate pathway analysis (Figure 4) of the bee metagenome revealed the variation in enzyme distribution and highlighted the change in metabolic shifts in the honeybee gut microbiome in response to nutritional availability. Several key glycolytic enzymes were found exclusively in the feeding condition, suggesting an active glycolysis pathway that facilitated the efficient breakdown of glucose for energy production (Figure 4). In non-feeding conditions function of gluconolactonase (EC 3.1.1.17) was found to be impeccable for the conversion of ꞵ-D-glucose. Adding on to that, in the case of feeding conditions, the same cycle was facilitated by dehydrogenases (EC 1.1.1.2.1.5). Comparing both conditions, the enzyme activity was pronouncedly elevated in favour of feeding. The transformation of D-glyceraldehyde into glycerate 2P in galactose metabolism is catalyzed by hexokinase (EC 2.7.16.5), thereby aiding in energy production.

On the other hand, energy production in the natural feeding category was mainly facilitated by phosphomevalonate kinases (EC 2.7.4.2.3). In accordance to that the D-glucosaminate metabolism was found out to be led by (EC 2.7.1.2.0.3 and EC 4.3.1.2.9) also known as glucokinase, is an enzyme that catalyzes the phosphorylation of glucose to glucose-6-phosphate, a key reaction in glucose metabolism, found in microorganisms and invertebrates.

### 3.7 Pyruvate pathway analysis

In the case of supplemental feeding, the enzymic activity was far more evident than in the non-feeding condition (Figure 5). The production of pyruvate from glycolysis was carried forward by (EC 4.1.1.4.9), which is the enzyme aldolase, specifically the aldolase of the type that catalyzes the reversible cleavage of fructose–1,6–bisphosphate into dihydroxyacetone phosphate and glyceraldehyde 3–phosphate.

EC 2.7.9.2, also known as pyruvate, water dikinase or phosphoenolpyruvate synthase, catalyzes the reaction that converts pyruvate, ATP, and water into phosphoenolpyruvate, AMP, and inorganic phosphate. Further adding up to that, the synthesis of amino acids like leucine, lysine and butanoate was only observed in feeding conditions. Lysin biosynthesis was governed by EC 2.3.3.1.4, also known as citrate (Si) synthase. It is an enzyme that catalyzes the first step in the citric acid cycle (Krebs cycle), converting oxaloacetate and acetyl-CoA into citrate in the feeding condition. Another enzyme, EC 2.3.1.9, known as CoA C-acetyltransferase or acetoacetyl-CoA thiolase, catalyzes the condensation of two acetyl-CoA molecules into acetoacetyl-CoA, a key step in ketone body and isoprenoid biosynthesis. It also played an intermediate role in butanoate synthesis in feeding conditions.

Alternately, in the non-feeding condition, the oxaloacetate formation from pyruvate was led by enzyme pyruvate carboxylase (EC 6.4.1.1). It is a biotin–dependent enzyme that catalyzes the ATP–dependent carboxylation of pyruvate to oxaloacetate, a crucial step in gluconeogenesis and other metabolic pathways. Another step is the propanoate metabolism or the breakdown of propionic acid, which involves several key steps. It starts with propionate’s conversion to propionyl-CoA, followed by carboxylation and isomerization, ultimately leading to succinyl-CoA, which can enter the citric acid cycle, was facilitated by pyruvate synthase or pyruvate: ferredoxin oxidoreductase (PFOR). The responsible enzyme for the above reaction is EC 1.2.7.1, which also catalyzes the oxidative decarboxylation of pyruvate to form acetyl-CoA and carbon dioxide, with ferredoxin acting as an electron acceptor.

### 3.8 Important KEGG and COG pathways in the metagenome

In the context of KEGG functional analysis, to characterize the gut microbiota of adult worker bees in two treatments, i.e., sucrose feeding and non–feeding, a comparative context has been represented in Figures 6A,B. There are approximately nine protein compounds which were found in both treatments, as shown in Figures 6A,B. Among those compounds, K01992, K02004, K01990, and K02003 are upregulated to a considerable extent in the feeding condition. The range varies between 2000 and 3,000 transcripts per million (TPM). In contrast, in non–feeding conditions, compounds like K01223 and K06147 are seen to be slightly upregulated to a range of 1,500–2000 TPM. The rest of the proteins did not hold a significant value and are seen to be down-regulated in both cases, namely, K15580, K02529, and K02015. Despite the presence of these similar proteins, the concentration varied significantly in both conditions (Figures 6A,B). Our findings revealed that there is a vast difference between feeding (3,000 TPM) and non-feeding (1,500 TPM) populations (Figures 6A,B).

### 3.9 COG pathways in the metagenome

The heat map displayed the expression levels of key Clusters of Orthologous Groups (COGs) in the metagenome across two samples, with expression measured in Transcripts Per Million (TPM) (Figure 6B). Our findings revealed that COG1132 showed the highest expression in non-feeding Sample (approximately 4,000 in TPM), indicating its significant presence in that sample. In contrast, feeding Sample exhibited relatively higher expression levels for COG1131 and COG1136, with values around 3,000 TPM (Figures 6C,D). COG1264 had the lowest expression across both samples, suggesting minimal involvement in the microbial processes of these environments. Moderate expression levels were observed for COG1609 and ENOG410XNMH in both feeding and non–feeding samples, with slight variations (Figures 6C,D). The differences in expression patterns indicated distinct functional activities between the feeding and non–feeding populations.

The present findings revealed that the cluster of orthologous genes, along with their proteins, is markedly upregulated in the non–feeding condition. Which is ultimately turning this adversity into an opportunity for further exploration regarding the possible causes.

## 4 Discussion

The present study characterizes the microbiota of *A. cerana indica* in sucrose-fed and non-fed conditions, which analyses the relative abundance and varied diversity of bacterial communities present in the gut and reveals the metabolic pathways in the metagenome. Analyzing the genomic diversity of microbial communities is challenging due to the high complexity in controlled sampling (Engel et al., 2012). However, the honey bee gut microbiota, with its low phylotype–level diversity, provides a suitable model system. In this context, metagenomics approaches have been used to explore the genomic diversity of this community in depth.

Our results implied that the variation in diversity was significant between the two groups. Ellegaard and Engel (2019) reported multiple comparison studies between *A. mellifera* and *A. indica.* His result clarified that the strain level diversity is higher in *A. mellifera* than *A. indica* in a natural diet. Feeding characteristics were the major deciding factor influencing the structure and function of the gut microbiomes of honey bees (Wang et al., 2020). An earlier study examined the microbiota configuration and function in representatives of different orders mediated by feeding characteristics to deeply understand the interaction between feeding conditions and the gut microbiota (Zheng et al., 2022). By exploring the metabolic pathways and enzymes, this study sheds light on how different microbes impacted bee metabolism, digestion and immunity, which aids in guiding strategies to enhance bee health and productivity.

The present results confirmed that the gut microbiome of *A. cerana indica* in two different treatments, i.e., with and without sucrose feeding, includes a core microbiome consisting of relatively higher numbers of Gram–positive bacterial species (Figures 1A–D). Our findings encompassed the most abundant bacterial community belonged to the phylum *Firmicutes*, particularly the class *Bacilli*, with *Lactobacillus* accounting for a significant chunk of the bacterial population that is shown in the taxonomy stacked bar charts (Figures 1C,D). Moreover, we found a significant presence of core species like *Gilliamella, Bacillus, Snodgrassella, Lactobacillus, Bombilactobacillus, Apibacter, Staphylococcus, Clostridium* and *Bifidobacterium* (Figures 1C,D). On the contrary, *Enterococcus, Enterobacter*, and *Lactiplantibacillus* were not detected in feeding samples of the *A.indica,* which was observed in non–feeding conditions (Baud et al., 2023). The bacterial load was markedly different in the tested samples. The possible driving force of these observed changes in bacterial loads and community composition in foragers could be dietary differences between the analyzed bee treatments. Anderson et al. (2014) opined that three types of *A. mellifera* bees, i.e., winter, nurse and foragers, have reported the same kind of core community diversity in three different seasons, having diverse feeding patterns.

*Lactobacillus, Bombilactobacillus*, and *Bifidobacterium* are indispensable members of the bee gut microbiome, thriving in the honeybee digestive system and helping in metabolizing complex nutrients to harness its nutrients (Raymann and Moran, 2018). Interestingly, the genus *Bombilactobacillus* was detected in both treatments of the current study. This genus previously classified under *Lactobacillus* species associated and identified in the stomach and hindgut with social bees (*Apis mellifera* and *A. indica*) has recently been reclassified as a distinct genus (Zheng et al., 2020). Remarkably, *Lactobacillus* is often found in honey, bee products and bee foraging crops. It has been reassigned from its original classification under *Lactobacillus* to the genus *Apilactobacillus*. Notably, our results are consistent with those of Ellegaard et al. (2020), who also reported the vast strain level difference in the core microbiome in both *A. mellifera* and *A. cerana indica*. Current results also found members of *γ*–proteobacteria, such as *Snodgrassella* and *Gilliamella*. Interestingly, in the case of *Snodgrassella,* the abundance barely changed across worker bees of two diet treatments in the experiment. This proclaimed that the colonization of *Snodgrassella* is neither modulated by the dietary supplements nor by the physicochemical environment in the gut or the abundance of other community members. A probable reason could be that the niche of *Snodgrassella* relays on the host rather than the diet because it selectively colonizes the epithelial lining of the ileum, which presents a physically restricted niche (Zheng et al., 2017).

While these microbial species appeared in lesser abundance in both treatment groups, emerging research indicates that even the less prevalent members play essential roles in the gut microbiome of *Apis* species (Tola et al., 2020). Comparable observations regarding the core gut microbiota of *Apis mellifera* have been documented by Ge et al. (2021), which supported our findings that *A. cerana indica* individuals from the exact location but exposed to different treatments harbored diverse microbial strains. In the feeding bees, the number of genera was lower than the non–feeding group; however, the microbial densities remained balanced and represented the core gut community typical of honey bees. Notably, *Enterococcus* and *Enterobacter* were particularly dominant in the non–feeding condition, though their distribution was uneven. These findings suggest that diet significantly influences the microbial composition observed in worker bees. In addition to diet, seasonal variations and environmental factors also play a considerable role (Kešnerová et al., 2020). These microbial changes often align with nutritional shifts, supporting the widely held view that dietary habits are a significant factor shaping microbial communities in various animal species (Sonnenburg et al., 2016; Sommer et al., 2016).

A larger volume of ingested food in honeybees likely enhances the gut’s capacity to support microbial populations. Additionally, pollen provides a more nutrient–dense diet compared to nectar, honey or sucrose, offering a broader array of metabolic niches for gut bacteria to inhabit (D’Alvise et al., 2018). These factors together may explain the higher bacterial loads observed in bees consuming pollen versus those fed only sugar water. Our results showed a rise in both bacterial abundance and species richness in worker bees that consumed pollen. Supporting this, studies in mice and flies by Erkosar et al. (2018) and Llewellyn et al. (2018) indicate that diets richer in natural nutrients, particularly protein, are linked to increased microbial abundance, though sometimes accompanied by reduced *α*–diversity. Furthermore, the *Enterococcus* and *Enterobacter*, bacteria were less abundant in bees than in other insects (Hystad et al., 2017). These findings suggest that in addition to diet, other elements are likely to influence the microbial community differences observed between feeding and non–feeding bees.

Indeed, in a prior metagenomics study in *A. mellifera*, conducted by Steinmann et al. (2015), it was shown that gut bacteria have a lower average population when fed with high fructose corn syrup as compared to those who were fed with normal honey, pollen and nectar. Present findings revealed that the gut microbiota of worker bees undergoes characteristic shifts in feeding as well as non–feeding. These changes may have significant consequences for the host.

The KEGG Pathway database serves as an essential tool in metabolomics and other multi–omics studies, particularly for interpreting complex metabolic pathways that are associated with genes. In the context of metabolomics data analysis, it offers comprehensive insights into metabolic functions and gene interactions. Our metagenomics investigation further identified functionally relevant gene profiles linked to functional enzymes, offering a broader perspective on microbial diversity and community dynamics beyond conventional 16S rRNA and *ITS* analyses. Notably, the current results revealed the presence of several enzymes within bacterial populations that are integral to host immune defense, carbohydrate metabolism, and energy homeostasis. For instance, enzymes such as β–glucosidase and ꞵ–D–glucose were detected across both treatment groups that played significant roles in supporting the host’s metabolic and defense mechanisms. Comparable findings in *Aphis pomi* demonstrated elevated enzymatic activity in response to temperature fluctuations (Dampc et al., 2020). While this observation was specific to *A. pomi*, it is reasonable to hypothesize a similar mechanism in honeybees, where these enzymes may be activated as part of an adaptive immune response to thermal stress (Dampc et al., 2020). These enzyme activity patterns underscore how nutritional inputs can shape metabolic shifts in the honeybee gut microbiota.

From a functional point of view, the honey bee proteins found in the co–immunoprecipitated complexes were mostly related to translation, catabolic functions, cytoskeleton structure, and protein transport (Becchimanzi et al., 2025). Close inspection of this protein list revealed the presence of several enzymes that are well-known components of the main pathways of central carbohydrate metabolism. In the current findings, three major pathways have been identified for core energy production. In both treatment cycles like glycolysis for catalyzing treatment cycles, glycolysis is used to catalyze the phosphorylation of glucose into glucose–6–phosphate, marking the first step in glycolysis. The intermediate steps consist of these enzymes like glyceraldehyde–3–phosphate dehydrogenase, phosphoglycerate mutase, and ATP–dependent 6–phosphofructokinase to carry forward this reaction. The tricarboxylic acid cycle and pentose phosphate pathway accommodate enzymes like citrate synthase, isocitrate dehydrogenase, oxoglutarate dehydrogenase and lastly 6–phosphogluconate dehydrogenase for energy production. These reactions catalyze the phosphorylation of (R)–5–phosphomevalonate to (R)–5–diphosphomevalonate using ATP, a reaction intermediate in the biosynthesis of isoprenoids, which are precursors for many important molecules like sterols and terpenes in the isoprenoid biosynthesis pathway. Most of the proteins that have been identified are found to be an inevitable part of ATP binding, and thereby, their presence and upregulation lead to major energy production (Bekele et al., 2025). Our findings are aligned with Becchimanzi et al. (2025), who also have established similar enzymes and pathways for *Apis mellifera.* These findings demonstrated a clear metabolic adaptation in the honeybee gut microbiome in response to feeding conditions. The feeding condition promoted glycolysis, supporting efficient ATP production and energy utilization, whereas the non–feeding condition triggered a metabolic shift toward gluconeogenesis and fermentation, ensuring microbial survival under nutrient scarcity. The observed differences in enzyme presence provided critical insights into how the honeybee gut microbiota dynamically adjusted its metabolism based on the availability of dietary resources.

Pentose phosphate pathway is an alternative pathway of glucose catabolism, which is mainly regulated by glucose–6–phosphate dehydrogenase (*G6PD*), 6–phosphogluconate dehydrogenase (*6PGD*), transketolase (*TKT*) and transaldolase 1 (*TAL*), (Ge et al., 2020). Several key glycolytic enzymes were found exclusively in the feeding condition, suggesting an active glycolysis pathway facilitating glucose’s efficient breakdown for energy production. In non–feeding conditions, the conversion of ꞵ–D–Glucose into D–Glucono–1,5 lactone further gave rise to D–Gluconate the whole process is called pentose and glucuronate interconversion which was facilitated by gluconolactonase. Metabolic genes such as (*G6PD KTK, TAL* etc.) include gluconolactonase, which may allow for the deacidification of royal jelly (Li et al., 2007; Furusawa et al., 2008; Sagona et al., 2015), and two copies of GMD, a gene that plays a role in the metabolism of mannose and fructose, components of nectar and honey (Freeman and Worthington, 1985; Smith and Newton, 2020).

Phosphomevalonate kinases are found only in the sucrose feeding condition, which is essential for isoprenoids, which are precursors for many important molecules like sterols and terpenes in the isoprenoid biosynthesis pathway. We identified several enzymes of the mevalonate pathway as well as two enzymes, namely gluconolactonase and hexokinase, involved in the sesquiterpenoid backbone biosynthesis. On the other hand, energy production in the natural feeding category was mainly from purine, pyrimidine, and histidine metabolism, and the responsible enzymes are phosphomevalonate kinases (EC 2.7.4.2.3). Similarly, the González-Caballero et al. (2014) depicted that the pheromone gland proteome includes enzymes of the mevalonate pathway also involved in sesquiterpenoid biosynthesis and proteins associated with pheromone production. The proteins identified in the pheromone gland of *L. longipalpis* provide interesting molecule candidates for pheromone production in this insect. This was validated by Vandermoten et al. (2009). In addition, they also found twelve proteins that are functionally related to the mevalonate pathway or have been reported as involved in pheromone or odor metabolism such as glutaminyl–tRNA synthetase, phosphoglycerate kinase, succinyl Co–A synthetase alpha subunit, transketolase, glutathione S transferases and cytochrome family proteins in insects.

The function of Glucokinase observed in our study is serves in hemolymph as a vital circulatory fluid throughout the honey bee’s body, playing a central role in transporting hormones and small–molecule regulatory factors that are crucial for regulating honey bee development. This is consistent with the experiment conducted by Li et al. (2025), where metabolomics was employed to investigate differences in the composition of hemolymph metabolites in larvae of worker bees (*Apis mellifera*) across three physiological developmental stages. After analyzing the metabolites of the three physiological stages and comparing the differences, an increase in up–regulated differential metabolites was found. This indicates the necessity for the hemolymph of pupae stage larvae to mobilize a more significant number of metabolites to fulfil the requirements of larval pupation and set the stage for subsequent emergence from cells. The linoleic acid, which is also observed in this pathway, involves metabolism in four steps: oxidation, assembly, transport, and clearance. Initially, fatty acids undergo oxidation in the insect fat body to be converted into linoleic acid, which is then released into the hemolymph. Subsequently, the hemolymph transports linoleic acid to target cells (Song et al., 2022).

The most numerous functional groups of proteins were enzymes and ribosomal proteins. These functional protein groups indicate an intense period of larval development, during which many new biomolecules are synthesized. This stage requires large amounts of energy, resulting in the presence of many enzymes involved in energy–producing metabolic pathways (Figures 6C,D). In our study, it has been established that the production of pyruvate was carried forward by the enzyme aldolase (EC 4.1.1.4.9). Specifically, aldolase of the type that catalyzes the reversible cleavage of fructose–1,6–bisphosphate into dihydroxyacetone phosphate and glyceraldehyde 3–phosphate. Insects, like other organisms, utilize pyruvate metabolism as a central hub for energy production and other metabolic processes, with pyruvate being a key intermediate in glycolysis and a precursor for various pathways like the tricarboxylic acid cycle (TCA) and gluconeogenesis (Ghani et al., 2024). Among the proteins that have been identified in our study were those involved in metabolic pathways that lead to the production of energy (da Silva et al., 2023). Likewise, Matuszewska–Mach et al. (2024) carried out a study regarding the nutritional value of honeybee larvae (*Apis mellifera*) through proteomic profiling and opined that the metabolic processes namely glycolysis (fructose–bisphosphate aldolase, glyceraldehyde–3–phosphate dehydrogenase), glycogenolysis (glycogen phosphorylase–like), and citric acid cycle (ATP–citrate synthase) are essential for growth and development of that honey bee. In addition, they also found out that many of the identified enzymes are involved in the synthesis or metabolism of endogenous compounds like lipids, carbohydrates, amino acids, peptides, proteins, and others. These pathways may also lead directly or indirectly to energy production in cells. It has been observed in feeding conditions that in glyoxylate metabolism, when malate is converted into oxaloacetate in the presence of enzyme malate dehydrogenase (quinone) EC 1.1.5.4, is an enzyme that catalyzes the oxidation of (S)–malate to oxaloacetate, with a quinone acting as an electron acceptor.

The comparative metagenomics analysis, particularly through KEGG functional annotation and COG classification, provided key insights into how artificial feeding influenced microbial community functions in the bee gut. In the context of KEGG analysis, the presence of common protein compounds in both treatments suggested a conserved core microbial functionality; however, their differential expression levels highlighted the influence of diet on microbial activity. Proteins such as K02003, K01992, K02004, and K01990 were significantly upregulated in sucrose–fed bees. These proteins, primarily belonging to the ATP–binding cassette (ABC) transporter family, play essential roles in nutrient uptake, xenobiotic detoxification, and cellular defence mechanisms. The elevated expression of these transporters in the feeding group indicated an increased metabolic demand and possibly an adaptive response to process the high–energy sucrose input. These findings align with earlier reports where ABC transporters were implicated in microbial resilience and cellular regulation under altered nutrient conditions (Bekele et al., 2025; Kim et al., 2020). On the other hand, proteins such as K01223 and K06147 were modestly upregulated in the non–feeding group, reflecting potentially higher activity in metabolic pathways associated with stress adaptation or the breakdown of complex natural diets (Zhang et al., 2024). The variation in expression between common KEGG orthologs across the two treatments demonstrated the honey bee gut microbiome’s dynamic nature and responsiveness to dietary modifications.

The heat map of COG pathways revealed the higher expression of COG1132 in the non–feeding group, which suggested enhanced activity of metabolic or stress response pathways, as this COG is often associated with cellular transport and energy metabolism. In the sucrose–fed group, the elevated expression of COG1131 and COG1136 could imply increased genetic information processing activities, including DNA replication and repair mechanisms (Gottschalk et al., 2006; Allen et al., 2009). The low expression of COG1264 in both samples may reflect the limited involvement of its associated functions, possibly due to the absence of specific environmental triggers or microbial populations responsible for its expression (Kumar Mondal et al., 2017). The variation in COG expression levels across the samples indicated differences in microbial community composition or functional responses to environmental factors.

COG1132 represents an ABC-type multidrug transport system, ATPase. It is a component of a larger system involved in the transport of various molecules across cell membranes. COG1132 ABC-type multidrug transport system, ATPase and permease. ABC transporters are involved in the movement of a wide range of substances, including sugars, lipids, peptides, heavy metals, and drugs. These components also play significant role in defense mechanisms, (gene- *Brucella*GL000067, protein-BMEII0250, Bme). What we also found out was COG1609 (gene -*Brucella*GL000134, protein-BMEII0312, Bme), a transcriptional regulator. Along with this, COG1136 otherwise known as LolD, is an ATPase component of the ABC-type lipoprotein export system, specifically the LolCDE complex. This is involved in the translocation of mature outer membrane-directed lipoproteins from the inner membrane to the periplasmic chaperone LolA. These proteins are covalently linked to a lipid molecule, which allows them to be anchored to the outer membrane of bacteria. LolD is an ATPase component of the LolCDE complex and is involved in the formation of the LolA-lipoprotein complex. The LolCDE complex, including LolD, facilitates the transfer of lipoproteins from the inner membrane to the periplasm, where LolA binds to the lipoprotein and helps in its translocation to the outer membrane. COG1131- (gene-*Brucella*GL000642, protein-BMEII0802).

We hypothesize that supplemental sucrose feeding significantly alters the gut microbial composition and functional potential in *Apis cerana indica*. Moreover, it has a major influence on key metabolic pathways, including KEGG glycolysis, pentose phosphate, pyruvate and COG pathways in the metagenome. The diverse composition and function of the gut microbiota in *Apis cerana indica are influenced by food supplements associated with distinct living conditions. This study explored the key microbial gut status and functional genes in Apis cerana indica under sucrose supplementation. The result found* sucrose-fed bees harboring higher microbial diversity than unfed bees, highlighting nutritional influence on microbial richness. Our findings revealed *Bacillus* were enriched in feeding as compared to non-feeding samples. Conversely, *Enterococcus* was dominant in non-feeding groups. The present finding focused on enzymatic activity and metabolic functionality in feeding and non-feeding conditions of *Apis cerana indica*. This enumerates the importance of sucrose feeding influencing the gut microbiota in *Apis cerana indica populations.*

## 5 Conclusion

The comprehensive metagenomic profiling of *Apis cerana indica* gut microbiota revealed distinct taxonomic, functional, and metabolic variations between sucrose-feeding and non-feeding worker bees. Sucrose supplementation exhibited a more microbial diversity and balanced community, as indicated by a higher Shannon (2.59) and Simpson (0.87) indices compared to non-feeding workers. The core gut microbiota represented 12 genera, such as *Bacillus*, *Gilliamella*, *Bifidobacterium*, and *Lactobacillus,* dominating in feeding bees under sucrose supplementation, whereas *Enterococcus* and *Enterobacter* were enriched in non-feeding conditions. KEGG and COG suggested that the sucrose supplementation elevated glycolysis, pyruvate metabolism, and amino acid biosynthesis, reflecting enhanced energy production and metabolic activity. On the contrary, non-feeding conditions relied on gluconeogenesis and fermentation pathways, indicative of metabolic stress adaptation. The Anv’o analysis revealed genus-specific variations, notable enrichment of *Bacillus* under sucrose supplement, and *Enterococcus* in non-feeding conditions. Overall, our findings establish a sucrose dietary supplement significantly shapes the gut microbial structure and metabolic functions of *A. cerena indica,* underscoring the critical gut health of honeybees. The encouraging results providing a foundation in developing targeted probiotic formulation and optimized feeding strategies to enhance gut health, immunity, and overall colony resilience in *A. cerena indica*. Further studies integrating mega-transcriptomics and metabolomics would elucidate host-microbiome interaction under varying nutritional and environmental conditions.

## Data Availability

The datasets presented in this study can be found in online repositories. The names of the repository/repositories and accession number(s) can be found in the article/Supplementary material.
