# Supplementary material for: Sucrose supplementation influences gut microbial diversity and functional shifts in Apis cerana indica
Source: Front Microbiol. 2026 Jan 22;16:1733283. doi: 10.3389/fmicb.2025.1733283 (PMC12908922; doi:10.3389/fmicb.2025.1733283)
Supplement: Supplementary file 1 [file Table_1.docx]

**Supplementary file**

**Table 1: Contig statistics of Gut microbiota**

| Particulars | Assembly |
| --- | --- |
| Number of contigs | 147146 |
| Total length | 153085914.00 |
| Longest contig | 615154 |
| Shortest contig | 200 |
| N50 (total no. of sequences that makes 50 percent of the genome) | 2514 |
| N90 (total no. of sequences that makes 90 percent of the genome) | 368 |
| Contigs at superkingdom (k) rank | 87292 (59.3%), in 4 superkingdoms |
| Contigs at phylum (p) rank | 85049 (57.8%), in 22 phyla |
| Contigs at class (c) rank | 84353 (57.3%), in 37 classes |
| Contigs at order (o) rank | 82025 (55.7%), in 89 orders |
| Contigs at family (f) rank | 76514 (52.0%), in 111 families |
| Contigs at genus (g) rank | 74369 (50.5%), in 203 genera |
| Contigs at species (s) rank | 15880 (10.8%), in 192 species |
| Congruent | 146551 (99.6%) |
| Disparity >0 | 596 (0.4%) |
| Disparity >= 0.25 | 445 (0.3%) |

**Supplementary Table S1: ORF (Open reading frame) Statistics of Gut microbiota**

| **Specifications** | **Feeding** | **Non-feeding** |
| --- | --- | --- |
| Number of ORFs | 175842 | 187705 |
| Number of rRNAs | 309 | 312 |
| Number of tRNAs/tmRNAs | 1453 | 1536 |
| ORFs by Aragorn | 1453 | 1536 |
| ORFs by Prodigal | 174080 | 185857 |
| ORFs by barrnap | 309 | 312 |
| Orphans (no hits) | 29530 | 32659 |
| No tax assigned (with hits) | 2356 | 2324 |
| KEGG annotations | 77453 | 86090 |
| COG annotations | 103279 | 113693 |
| Pfam annotations | 75760 | 86099 |

**Supplementary Table S2: Annotation details of Gut microbiota**

|  | **Assembly** | **Sample1(feeding)** | **Sample2(non-feeding)** |
| --- | --- | --- | --- |
| Number of reads | 104718228 | 58684264 | 46033964 |
| Number of bases | 16614036170 | 9311782472.00 | 7302253698.00 |

**Supplementary Table S3: Bins statistics of Gut microbiota**

| **Particulars** | **DAS** |
| --- | --- |
| Number of bins | 31 |
| Complete >= 50% | 23 |
| Complete >= 75% | 17 |
| Complete >= 90% | 13 |
| Contamination < 10% | 24 |
| Contamination >= 50% | 3 |
| Congruent bins | 10 |
| Disparity >0 | 21 |
| Disparity >= 0.25 | 8 |
| Hi-qual bins (>90% complete,<10% contam) | 10 |
| Good-qual bins (>75% complete,<10% contam) | 11 |
